# Patient Satisfaction with private Physiotherapy for musculoskeletal Pain

**DOI:** 10.1186/1471-2474-9-50

**Published:** 2008-04-15

**Authors:** Sarah N Casserley-Feeney, Martin Phelan, Fionnuala Duffy, Susan Roush, Melinda C Cairns, Deirdre A Hurley

**Affiliations:** 1 School of Physiotherapy & Performance Science, University College Dublin, Ireland; 2 School of Health & Emergency Professions, University of Hertfordshire, UK; 3 Physical Therapy Department, University of Rhode Island, USA

## Abstract

**Background:**

Despite emphasis on patient centred healthcare, healthcare professionals have been slow to use validated measurements of patient satisfaction in physiotherapy practice. The aim of this cross sectional survey was to measure patient satisfaction with private physiotherapy in Ireland, for patients with musculoskeletal pain, using a previously validated survey instrument.

**Methods:**

A multidimensional patient satisfaction questionnaire 'PTOPS', which assesses patient satisfaction with outpatient physiotherapy treatment, was translated from American English to European English, and relevant demographic and global satisfaction items were included. This was then circulated to patients with musculoskeletal pain (n = 240) for anonymous completion and return to the research team. Data were analysed using the Statistical Package for the Social Sciences (SPSS, v.12).

**Results:**

In total 55% (n = 131/240) of questionnaires were returned. Just over half of the respondents were male (53.4%, n = 70), with a mean age (SD) of 37.7 years (12.4), and had previous experience of physiotherapy (65.6%, n = 86). The most common site of musculoskeletal pain was spinal (51.5% n = 66). The mean (SD) number of treatments was 8.3 (8.3), at a mean total cost (SD) of €350.2 (€322.8). The 'PTOPS' questionnaire categorised and scored satisfaction items under four domains, Enhancer, Detractor, Location and Cost. The mean score (SD), optimum score, and scoring range for each domain were: 'Enhancer' 41.2 (3.8), 50, 10–50; 'Detractor' 19.4 (4.4), 10, 10–50; 'Location' 28.0 (4.1), 35, 7–35; 'Cost' 18.9 (2.8), 7, 7–35. "Overall satisfaction with physiotherapy experience" was scored on a five-point scale "excellent to poor", with a modal response of "Very Good" (42%; n = 55).

**Conclusion:**

This study measured patient satisfaction with private physiotherapy treatment for musculoskeletal pain in Ireland using a previously validated outcome measure and provides a template for future studies of this increasingly important topic. Results demonstrated high levels of satisfaction with all components of physiotherapy treatment, except cost, and provided valuable patient feedback regarding their physiotherapy treatment for musculoskeletal pain. Results can be used by physiotherapists to improve future patient experiences with a view to improving patient attendance and compliance with physiotherapy treatment protocols for patients with musculoskeletal pain.

## Background

Traditionally, consumer satisfaction has been afforded a high level of importance in commercial and market research. More recently, there has been a growing interest in the measurement of patient satisfaction in healthcare research, demonstrating a move towards patient centred care as recommended by the Department of Health [1,2]. Patient satisfaction surveys provide several benefits for healthcare professionals. They can be used to measure the success of delivering information [3], and to predict patient re-attendance and compliance with treatment [4,5], which is particularly relevant in the management of musculoskeletal problems where compliance with an 'exercise programme and/or a 'medication regime' are common interventions. There is mixed opinion in the literature regarding whether or not satisfaction levels are a reflection of the quality of healthcare [6], but the consensus is that patient satisfaction is reflective of the patient's perception of the quality of the healthcare they receive [7]. Nonetheless, patient feedback can be used systematically to improve methods of providing health care, such as the length of a patient appointment or arrangements for flexible opening times at a clinic [8]. Furthermore, patient satisfaction may provide the only discerning outcome measure in a study, as demonstrated by Seferlis et al [9], who reported similar clinical outcomes for three types of conservative treatment in a randomised controlled of low back pain (LBP), but a significantly higher level of satisfaction for the group receiving manual therapy, justifying future preference for this approach.

Internationally, musculoskeletal injuries have a prevalence of 60.3% to 68% [10], with the majority of care (93.1%), provided in the primary care setting by General Practitioner's (GP) and physiotherapists [11].

In Ireland, outpatient physiotherapy services for musculoskeletal injuries are delivered predominantly in private physiotherapy clinics where waiting times are shorter than their public counterparts [12], and musculoskeletal injuries account for the majority of private physiotherapy consultations workload [13]. While the Chartered Society of Physiotherapy (CSP, UK) has included patient feedback questionnaires in their Core Standards of practice [14], they have not provided any validated outcome to measure patient satisfaction with physiotherapy treatment and the majority of existing literature regarding patient satisfaction with physiotherapy is based on US populations, where differences in healthcare systems between there and Europe, specifically Ireland and the UK, make international comparisons difficult. Furthermore, many of the measurement tools used in previous studies of patient satisfaction with physiotherapy treatment have unclear psychometric properties regarding validity and reliability [6,15]. Although, no single methodology, for the measurement of satisfaction is recommended over another, most studies use self report questionnaires [16,17], which are less expensive, less time consuming, and have less potential bias towards false high scores than interviewer administered questionnaires [6,16].

One approach that has been used to measure satisfaction with physiotherapy, has been to use instruments designed for other health related disciplines [18], or to use a general patient satisfaction questionnaire [19,20]. General patient satisfaction questionnaires allow for greater comparability between a wide variety of healthcare disciplines, patient conditions or healthcare settings [16,19-22], which can provide a broad overview of issues that may require attention. However, the unique aspects of care related to outpatient physiotherapy, such as the need for longer treatment sessions than those of a typical doctor's visit for example, require a different, discipline specific scale. This is also true of healthcare setting, where the aspects of care related to inpatient or outpatient differ. Thus findings specific to a particular discipline or healthcare setting may be less obvious when more general questionnaires are used, limiting their discipline specificity [6,15,16].

In measuring patient satisfaction with physiotherapy, one must be clear regarding the parameter they wish to measure – 'patient satisfaction with physiotherapy treatment' or 'patient satisfaction with outcome', the current study wished to investigate the former. Both of these concepts are separate entities, independent of each other, and are influenced by different domains or factors [23]. Hudak and Wright [6] suggest that patient 'satisfaction with outcome' relates to the results of treatment, whereas 'satisfaction with care' reflects the service the patient received during the course of care. Such distinction seems especially relevant for patients who are satisfied with various treatment domains (i.e. access, interpersonal factors, and cost) but remain dissatisfied with their resultant ongoing symptoms. Furthermore it is now accepted that 'patient satisfaction with physiotherapy' is a multidimensional rather than a uni-dimensional parameter [5,6,24-27]. Uni-dimensional measures of patient satisfaction obviously provide a quick and easy means of measuring patient satisfaction, but provide no information regarding which aspects of a service a patients may have been particularly satisfied or dissatisfied with and tend to provide high satisfaction levels that are likely to be false positives [6]. Although no definitive set of dimensions or domains for 'patient satisfaction with physiotherapy treatment' exists, May [15] recommends patient involvement in the definition of these domains to increase the construct validity of a questionnaire.

A literature review was undertaken by three of the researchers (SCF, MP and FD) to identify a suitable questionnaire to measure patient satisfaction with physiotherapy for musculoskeletal pain. The clinical research databases PubMed, Cinahl, BMJ Journals, BioMedCentral, Embase, PsycInfo, PEDro and MEDLINE were searched between January and March 2007. The search terms 'Patient Satisfaction; Patient Satisfaction AND Physiotherapy; Patient Satisfaction AND low back pain; Patient Satisfaction AND musculoskeletal pain' were used in these databases, which identified numerous articles. Review articles or those which were part of symposia relating to the measurement of patient satisfaction were ordered according to publication date, and review of these articles provided a framework of criteria to guide the researcher in the identification of a suitable questionnaire (Table 1). [6,17-19,29] Thereafter, articles that measured "patient satisfaction with physiotherapy" in patients with LBP or musculoskeletal pain were deemed highly relevant, and those which measured patient satisfaction with physiotherapy in other patient populations were deemed relevant. These articles provided numerous questionnaires that measured patient satisfaction with physiotherapy. These questionnaires were evaluated to determine how well they adhered to the guidelines for a well developed questionnaire provided from the initial literature review (Table 1). Thus based on the recommendations of several authors [6,8,17,18,29], and considering the populations and locations in which they were developed and tested, The Physical Therapy Outpatient Satisfaction survey (PTOPS) [24], which had undergone multiphase psychometric testing to confirm it's validity and reliability, was finally selected as the survey instrument.

**Table 1:** Evaluation of Patient Satisfaction with Physiotherapy Questionnaires

	**Evaluation Criteria for Questionnaire (based on literature review)**				
	^a^Multidimensional	Self completed by patient	Likert Scale (≥ 5 levels)	^b^Negative Phrasing used	^c^Psychometric data available

Roush & Sonstroem (1999)*	Yes	Yes	Yes	Yes	Yes
**Goldstein et al (2000)**	Yes	Yes	Yes	No	Yes
**Beattie et al (2002)**	Yes	Yes	Yes	Yes	Yes
**Monnin & Perneger (2002)**	Yes	Yes	Yes	No	Yes
**French (2003)**	Yes	Yes	Mixed	No	No
**George & Hirsch (2005)**	No	Yes	Yes	No	No

*Fulfils all the evaluation criteria for questionnaire suitability;

^a ^The publication reports on the processes of selection and evaluation of the multiple dimensions (concepts) that may influence patient satisfaction in the questionnaire;

^b ^Questionnaire includes negatively phrased statements to minimise response bias (e.g. "I do not feel that the physiotherapist valued my opinion");

^c ^Information regarding scale development, validity, and reliability of the questionnaire were undertaken and included in publication.

Having identified a suitable measure of satisfaction, the aim of the current study was to explore the determinants and levels of satisfaction of patients attending private physiotherapy for musculoskeletal pain in Ireland, using this pre-existing validated patient satisfaction questionnaire. The literature regarding the measurement of patient satisfaction with physiotherapy treatment is somewhat limited by quantity and by the lack of consistent methodologies or outcome measures. Nonetheless, previous studies have demonstrated high levels of patient satisfaction with other treatments for musculoskeletal pain [7,30-33]. Therefore it was hypothesised that similarly high levels of satisfaction with physiotherapy treatment for musculoskeletal conditions would be found in this exploratory study. Furthermore, four global scales hypothesised to reflect patient satisfaction were included [24] and it was hypothesised that results from this survey would demonstrate strong correlation with these measures. Finally, the literature suggests that patient satisfaction may be associated with patient characteristics such as age and diagnosis [16,34,35], and the authors hypothesised that similar associations would be reported in this study.

## Methods

A cross-sectional survey of patients attending private physiotherapy clinics in Ireland was undertaken between May 2005 and June 2006, and consisted of two phases – 1) identification and cross-cultural translation of the patient satisfaction questionnaire and 2) patient satisfaction cross-sectional postal survey.

### Selection and Cross-Cultural Translation of the Survey Instrument

Following literature review, The Physical Therapy OutPatient Satisfaction Survey [PTOPS, [24]] was selected as the 'patient satisfaction questionnaire'. This questionnaire contains 34 positively and negatively worded statements that are scored using five-point Likert scales ranging from "strongly disagree" to "strongly agree", which fall into four categories of outpatient satisfaction – Enhancers, Detractors, Location and Cost. Enhancers – relate to the positive aspects, the satisfiers of the physiotherapy experience – e.g. topics that enrich a patient's experience beyond a minimally acceptable level, Detractors – relate to a patient's basic physical and interpersonal needs that if present or not create positive or negative feelings, Location – relates to ease of locating and travelling to a clinic, and Cost – relates to monetary cost and perceived financial value. Low scores for the 'Detractor' and 'Cost' components, and high scores for the 'Enhancer' and 'Location' components of the PTOPS represent high levels of overall satisfaction respectively. This questionnaire was developed in the United States by Roush and Sonstroem [25], who undertook a 3-phase study, to identify the underlying components of outpatient satisfaction with physiotherapy and to develop a test that would yield reliable and valid measurements of these components. Three samples of outpatients (n = 177, 257, and 173 respectively), attending physiotherapy were recruited from 21 physiotherapy clinics. In phase 1 of the study, principal component analyses (PCA), reliability checks, and correlations with social desirability scales were used to reduce a pool of 98 items to 32 items. These analyses identified a five component model of outpatient satisfaction in physical therapy. The phase 2 PCA, with a revised pool of 48 items, indicated that four rather than five domains presented the best model giving rise to the 34-item PTOPS, which was supported by factor analyses conducted with phase 2 and phase 3 data which confirmed the independence of the domains providing evidence for the internal validity of the PTOPS scores. External validity was evaluated against global high/low satisfaction and correctly classified 93.8% of subjects and reliability testing reported values of 0.71 to 0.87 [24].

A team of three independent Irish physiotherapists experienced in musculoskeletal management translated the original PTOPS from American English into a European English [36] version of the PTOPS. Hence, 'physical therapist' became 'physiotherapist', and 'parking lot' became 'car park'. Changes to the original questionnaire were collated by the Irish team leader (DH) and sent to the US team leader (SR) for retranslation, and original and final versions were compared to ensure that linguistic equivalence was achieved [28]. The US team also consisted of three independent physiotherapists experienced in musculoskeletal management. This translated 34 item PTOPS questionnaire was incorporated into the final survey instrument in the current study which comprised of three sections:

(i) four global measures of patient satisfaction. These global measures used 5-point rating scales (excellent, very good, good, fair, poor) and measured overall improvement, overall satisfaction with treatment, chances of returning to this clinic and chances of recommending this clinic to a friend.

(ii) the PTOPS questionnaire. The 34 items of the PTOPS questionnaire measured patient satisfaction with physiotherapy treatment only.

(iii) socio-demographic section. This recorded patient and treatment details, and included a comment section to allow the respondent to give feedback on the physiotherapists and the physiotherapy clinics.

This final survey instrument was named the EPTOPS (European Physiotherapy Treatment Out Patient Satisfaction Survey) to differentiate from the original instrument and was used in this cross-sectional survey.

### Data collection

Ethical approval was gained from the University of Hertfordshire Faculty of Health and Human Sciences, Radiography and Physiotherapy Ethics Committee.

The total study population comprised all the practices of members (n = 253) of the Irish Society of Chartered Physiotherapists in Private Practice (CPPP). All members of the CPPP were emailed in order to establish interest and recruit practices for the study. Whilst, the majority of private practitioners (n = 172) expressed interest in the study, only seventy three were willing to participate within the timeframe of the study and the majority of these were urban based. Consequently a convenience sample of privately owned chartered physiotherapy practices was selected, which represented diverse locations throughout the Republic of Ireland, including both urban and rural settings. It was aimed that a minimum of 100 completed questionnaires should be available for analysis. Based on an expected minimum response rate of 50%, and expecting each participating clinic to distribute 10 questionnaires, it was calculated that 20 clinics would be required to participate (n = 200 questionnaires). Thus allowing for a 50% clinic participation rate, a sample of convenience of 40 private physiotherapy clinics practices were mailed a letter and telephoned in person (FD & MP) regarding the aim of the study, of which 24 agreed to participate. Written consent from practice owners was then obtained to allow the inclusion of their clinics.

Two weeks prior to the data collection period, each of the 24 participating practices received one 'physiotherapist study pack' and ten 'patient study packs'. All practices were phoned by the researchers (FD & MP) to ensure that all study packs had been received and to answer any queries arising.

• Patient study packs included a patient introductory letter, a study information sheet, a copy of the survey instrument, a consent form, and a stamped addressed envelope (SAE).

• Physiotherapist study packs included a full copy of the patient study pack, an introductory letter and study information sheet including the patient recruitment guidelines protocol, and a clinic owner's consent form with SAE.

The recruitment protocol was provided to ensure standardisation of patient recruitment and reduce potential patient selection bias on the part of the physiotherapist. Physiotherapists and patients were provided with contact details of the researchers if additional information was required.

Subjects were eligible for inclusion if they were over 18 years, receiving treatment for a musculoskeletal condition, could read and understand English, and provided written informed consent. They were excluded if they did not meet the inclusion criteria or if their treatment had medicolegal implications. Patients were requested to return the completed questionnaire and a signed copy of the consent form in the provided Stamped Addressed Envelope directly to the researchers. It was clearly explained to all subjects that their care within the clinic would not be compromised if they did not participate in the study. Data collection was anonymous – subjects were asked not to identify themselves, their therapist, or the attending physiotherapy clinic. During the data collection period all clinics received a phone call to answer any queries and to promote a satisfactory response rate.

### Data Analysis

Data were coded, scored and logged into spreadsheets in the Statistical Package for the Social Sciences (SPSS: Version 12.0) by FD and MP, and checked for errors by SCF who screened a random selection of 25 data sets and score sheets prior to logging, and screened all datasets for errors using define variable and explore applications in SPSS after logging. Data analysis was conducted using SPSS. Simple descriptive statistics were used to explore respondent characteristics, mean summary satisfaction scores and the global ratings of satisfaction. Relationships between satisfaction domain scores and subject characteristics were investigated using non-parametric tests. Four regression analyses were taken, one for each of the four domain scores of the PTOPS questionnaire and the independent patient socio-demographic and health related baseline variables. The independent variables included whether the patient was male or female (1,0), had learned about the physiotherapist from their GP or another source (1,0), it was their first time to attend physiotherapy or not (1,0), they had a spinal or non spinal musculoskeletal problem (1,0), were married or other (1,0), held a professional qualification or not (1,0), were aged less than 38 years or older (0,1) and the total number of physiotherapy treatments.

External validity of the PTOPS questionnaires was examined using Pearson's correlation coefficient to test the correlations between the domain scores and each of the global scales.

Data obtained from the three open-ended questions were primarily descriptive, and themes were identified from these questions using Microsoft Excel XP.

## Results

### Survey Response Rate

Forty private physiotherapy practices were approached to participate in the study, of which 24 agreed. A total of 240 questionnaires (10 per practice) were distributed to eligible subjects, of which 131 were returned to the researchers (response rate = 55%).

Respondent Characteristics and Treatment Details

Respondents' characteristics showed that just over half were male (53.4%, n = 70), with a mean age (SD) of 37.7 years (12.4), married (56.5%, n = 74), Caucasian (100%, n = 131), employed (74.8%, n = 98), had completed secondary school (84.0%, n = 110), and held a higher degree or professional qualification (54.2%, n = 71) (Table 2). The most common reasons for attending physiotherapy were spinal pain (51.5%, n = 66), or lower limb (32.8% n = 42), particularly knee complaints (13.3%, n = 17). The majority of respondents had previous experience of physiotherapy (65.6%, n = 86). In relation to referral pathways, the majority of respondents (62.6%, n = 82) learnt about the relevant physiotherapy practice through friends or former patients of the clinic, while 26% (n = 34) were referred by their general practitioner (GP). Only 5.3% (n = 7) learnt of the physiotherapy clinic from the Golden Pages phone directory. Regarding treatment received, manual therapy was reported as the most common (93.9%, 123), while the majority also received advice/information (78.6%, n = 103), exercise therapy (77.9%, n = 102) and a home exercise programme (71%, n = 93). The mean (SD) number of treatments was 8.27 (8.28) at a mean total cost of treatments was €350.19 (€322.81), and mean cost of €41.00 (€8.36) per treatment. The majority of patients attending physiotherapy (80.9%, n = 106) self-funded their treatment costs. Other patients' fees were reimbursed by private health insurance companies (8.4%, n = 11), such as VHI or BUPA, or by their sports club (9.9%, n = 13).

**Table 2:** Respondents' characteristics, treatment details and PTOPS scores (n = 131).

**Category**	**n (valid percentage %)**
**Male**	70 (53.4)
**Mean Age yrs (SD)**	37.7 (1.44)
**Caucasian Race**	131 (100)
**Marital Status:**	
Married	74 (56.5)
Single	45 (34.4)
Widowed	6 (4.6)
Separated	3 (2.3)
Other	3 (2.3)
**Employment Status:**	
Employed	98 (74.8)
Homemaker	10 (7.6)
Student	10 (7.6)
Retired	9 (6.9)
Looking for first job	3 (2.3)
Unable to work due to sickness/disability	1 (0.8)
**First time to have physiotherapy Source of patient information:**	45 (34.4)
Friends/Former patients	82 (62.6)
General Practitioner (GP)	34 (26.0)
Telephone (Golden Pages)	7 (5.3)
Other	7 (5.3)
**Higher Degree/Professional Qualification:**	71 (54.8)
**Presenting problem:**	
Spinal	66 (51.5)
Upper limb:	25 (19.5)
Shoulder; Elbow; Hand/wrist	10 (7.8); 10 (7.8); 5 (3.9)
Lower Limb:	42 (32.8)
Hip; Knee; Ankle/Foot	10 (7.8); 17 (13.3); 15 (11.7)
Other	3 (2.3)
**Treatment modalities received:**	
Manual therapy	123 (93.9)
Advice/Education	103 (78.6)
Exercises	102 (77.9)
Home exercise programme (HEP)	93 (71)
Electrotherapy	87 (66.4)
Equipment (e.g. Brace/Insoles)	21 (16)
Cardiovascular Exercises (e.g. walking)	19 (14.5)
Other	3 (2.3)
**Satisfaction Domain – Mean (SD)**	
Enhancers	41.2 (3.8)
Detractors	19.4 (4.4)
Location	28.0 (4.1)
Cost	18.9 (2.8)

### Satisfaction Questionnaire scores

#### Satisfaction Scores

The mean (SD), optimum scores and scoring range for each of the four PTOPS dimensions are presented in Table 1 and display overall high levels of satisfaction with private physiotherapy in Ireland. The results for, i) Enhancers and ii) Location were comparable to the optimum scores, showing that patients were satisfied with these parameters, whilst respondents appeared 'very dissatisfied' with the Cost domain and 'somewhat dissatisfied' with Detractors (Table 2).

#### Influence of patient characteristics on satisfaction scores

Patient characteristics (gender, age, degree or professional qualification, educational status and previous physiotherapy treatment) were collapsed to create dichotomous variables, and the relationship between these characteristics and satisfaction scores for each domain were analysed using Mann Whitney U tests (Table 3). Results showed significant differences for four variables: gender and age groups for Detractor scores, those with and without degree or professional qualification for Location scores, and varying degrees of educational status and the Cost score. However, the Bonferroni correction for multiplicity (0.05/5 = 0.01) rendered these findings non significant, except for the Detractor score, where younger respondents (<38 yrs) were less satisfied. Furthermore, while results of the stepwise multiple regression analyses showed significantly higher satisfaction (higher Enhancer scores) for those with spinal complaints and significantly higher satisfaction (lower Detractor scores) with increasing age, there was no consistent association between any baseline socio-demographic or healthcare variable and all four domains. There was no significant association between the Location or Cost domains and any baseline socio-demographic or healthcare variables (Table 4) and independent variables accounted for between 4.0–9.6% of the variation in the satisfaction with physiotherapy treatment scores.

**Table 3:** Influence of patient characteristics on PTOPS domain scores (n = 131)

**Patient Characteristic**	**Median (IRQ)**		**Test Statistic^Ω^**	**p value**
	**Enhancer**			
Gender (Male: female)	41.0 (5.5)	41.0 (6.0)	1968.000	0.439
Age (<38: ≥ 38 years)	41.0 (6.0)	42.0 (5.3)	1995.000	0.518
First Physiotherapy (Yes: No)	40.0 (6.5)	41.0 (5.0)	1720.000	0.296
Qualification/Degree (Yes: No)	41.0 (5.0)	40.0 (7.0)	2117.000	0.952
Secondary School Educational (Not complete: Complete)	42.0 (6.5)	41 (6.0)	1027.000	0.420
				
	**Detractor**			
Gender (Male: female)	20.0 (5.0)	18.0 (5.0)	1634.000	0.020*
Age (<38:≥ 38 years)	21.0 (4.0)	18.0 (4.0)	1331.000	<0.001*β
First Physiotherapy (Yes: No)	19.0 (5.0)	19.0 (4.5)	1738.000	0.338
Qualification/Degree (Yes: No)	19.0 (5.0)	19.9 (5.0)	2016.000	0.597
Secondary School Educational (Not complete: Complete)	18.0 (6.0)	19.0 (5.0)	957.000	0.213
				
	**Location**			
Gender (Male: female)	28.0 (5.0)	28.0 (5.0)	2016.500	0.582
Age (<38: ≥ 38 years)	28.0 (5.0)	28.0 (4.0)	2094.500	0.851
First Physiotherapy (Yes: No)	28.0 (3.5)	28.0 (5.0)	1707.000	0.266
Qualification/Degree (Yes: No)	29.0 (4.0)	28.0 (6.0)	1686.500	0.039*
Secondary School Educational (< 6 years: ≥ 6 years)	28.0 (4.5)	28.0 (5.0)	1150.000	0.975
				
	**Cost**			
Gender (Male: female)	19.0 (4.0)	19.0(3.0)	1887.500	0.308
Age (<38: ≥ 38 years)	19.0 (4.0)	19.0 (3.0)	1913.000	0.379
First Physiotherapy (Yes: No)	19.0 (4.0)	19.0 (3.5)	1834.500	0.701
Qualification/Degree (Yes: No)	19.0 (4.0)	19.0 (4.0)	825.000	0.204
Secondary School Educational (< 6 years: ≥ 6 years)	17.0 (4.0)	19.0 (4.0)	819.500	0.038*

^Ω ^Mann Whitney U test; *Significant at p < 0.05; ^β^Significant at p < 0.01 (Bonferroni adjustment for multiplicity)

**Table 4:** Regression Analysis of PTOPS scores on baseline socio-demographic and healthcare variables (n = 131)

**Enhancer**								
**Overall Model**		**r**^2^	**F value**	**P value**				

		0.040	5.334	0.022				

**Predictor Variables**	**Injury Site**	**Age**	First time to attend PT*	**No. of PT sessions**	**Gender**	**Marital Status**	**Profession/Degree**	**GP referral to PT**

**Beta**	0.200	-0.022	-0.102	0.155	-0.047	-0.116	-0.041	0.031
**t-value**	2.314	-0.243	-1.182	1.784	-0.540	-1.340	-0.467	0.354
**significance**	0.022	0.808	0.240	0.077	0.590	0.183	0.641	0.724

**Detractor**								

**Overall Model**		**r**^2^	**F value**	**P value**				

		0.096	13.568	<0.001				

**Predictor Variables**	**Injury Site**	**Age**	First time to attend PT*	**Number of PT sessions**	**Gender**	**Marital Status**	**Profession/Degree**	**GP referral to PT**

**Beta**	-0.063	-0.310	0.110	-0.044	0.147	0.131	-0.093	-0.036
**t-value**	-0.718	-3.683	1.311	-0.517	1.707	1.415	-1.102	-0.425
**significance**	0.474	<0.001	0.192	0.606	0.090	0.159	0.273	0.671

**Location**								

**Overall Model**		**r**^2^	**F value**	**P value**				

		0.096	13.568	<0.001				

**Predictor Variables**	**Injury Site**	**Age**	First time to attend PT*	**Number of PT sessions**	**Gender**	**Marital Status**	**Profession/Degree**	**GP referral to PT**

**Beta**	0.123	-0.034	-0.079	0.111	0.109	0.053	0.170	-0.057
**t-value**	1.307	-0.323	-0.872	1.222	1.142	0.537	1.839	-0.622
**significance**	0.194	0.747	0.385	0.224	0.256	0.592	0.068	0.535

**Cost**								

**Overall Model**		**r**^2^	**F value**	**P value**				

		0.055	0.872	0.542				

**Predictor Variables**	**Injury Site**	**Age**	First time to attend PT*	**Number of PT sessions**	**Gender**	**Marital Status**	**Profession/Degree**	**GP referral to PT**

**Beta**	-0.048	-0.078	0.064	-0.013	0.111	0.129	0.149	-0.059
**t-value**	-0.502	-0.724	0.687	-0.143	1.140	1.279	1.584	-0.637
**significance**	0.617	0.470	0.494	0.886	0.256	0.203	0.116	0.525

* PT = Physiotherapy

#### Global Reponses

The following global statements were scored on a five-point Likert scale "excellent to poor", and the modal responses were:

Excellent (60.2%; n = 80): Chance of recommending practice to a friend.

Excellent (62.6%; n = 82): Chances of returning to this clinic.

Very Good (42%; n = 55): Overall Satisfaction with the physiotherapy experience.

Very Good (40.5%; n = 53): Overall rate of improvement.

**Figure 1:**
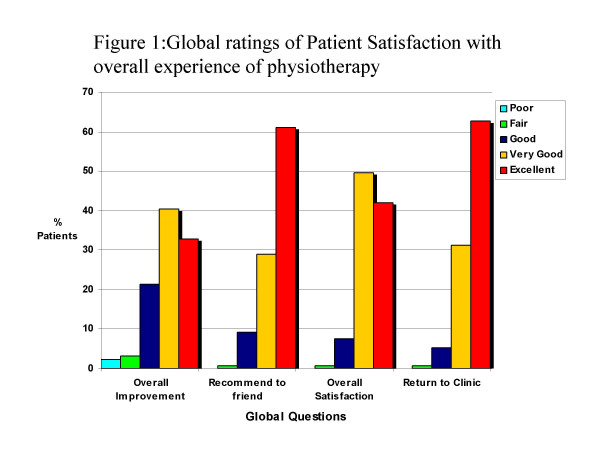
Global ratings of Patient Satisfaction with overall experience of physiotherapy.

These results demonstrate that patients were generally satisfied with the physiotherapy experience and outcome (Figure 1).

External validity of the PTOPS questionnaire was tested using Pearson's correlation coefficient, which examined the correlations between each of the domain scores and each of the four global scales. Results showed significant associations between all four global scales and Enhancer and Detractor scores. "Overall satisfaction with physiotherapy experience" was also significantly correlated with Cost, but there was no significant correlation between any of the global scales and the Location score (Table 5).

**Table 5:** Pearson's Correlation Coefficients for Global Responses and PTOPS domain scores (n = 131)

		**Enhancer**	**Detractor**	**Location**	**Cost**
**Overall improvement due to physiotherapy**	Pearson Correlation	0.431	-0.175	0.162	-0.153
	Sig. (2-tailed)	<0.001	0.046	0.065	0.082
**Chance of recommending clinic to family/friend**	Pearson Correlation	0.383	-0.243	-0.005	-0.131
	Sig. (2-tailed)	<0.001	0.005	0.952	0.139
**Overall satisfaction with physiotherapy experience**	Pearson Correlation	0.443	-0.298	0.069	-0.181
	Sig. (2-tailed)	<0.001	0.001	0.433	0.040
**Chance of returning to this clinic**	Pearson Correlation	0.459	-0.251	0.045	-0.158
	Sig. (2-tailed)	<0.001	0.004	0.607	0.073

#### Open ended questions

Finally, of the 131 completed questionnaires, 64.1% (n = 84) completed the 'clinic strength's question, 36.6% (n = 48) completed the 'clinic weakness' question and only 11.4% (n = 15) provided feedback on the 'questionnaire' (Table 6). The majority of positive feedback regarding the clinic related to the physiotherapists' characteristics and included comments such as 'helpful', 'knowledgeable' etc. The majority of negative feedback related to the clinic's location and standard of the premises, while other issues identified included a lack of privacy, lack of administration/support staff, and cost.

**Table 6:** Feedback from Open-Ended Questions

**Feedback Category**	**Frequency (n)**	**Valid Percentage (%)**
**Strengths of Clinic/Physiotherapist**	**84**	**100**
Friendly (helpful, caring, polite)	72	85.7
Professional (knowledgeable, skilful)	64	76.1
Flexible Opening Hours	30	35.7
Convenient Location	18	21.4
Staff (personal, approachable)	7	8.3
		
**Weaknesses of Clinic/Physiotherapist**	**48**	**100**
Location (parking, access via stairwells)	13	27.0
Premises (old premises, untidy)	12	25.0
Privacy	8	16.6
Too Busy (answering calls/doors)	4	8.3
Treatment Cost	3	6.2
		
**Survey Instrument Feedback**	**15**	**100**
Repetition of questions	9	60.0
Ambiguous	8	53.3
Time consuming	6	40.0

## Discussion

This study has measured patient satisfaction with private physiotherapy for musculoskeletal pain in Ireland for the first time, using a validated physiotherapy-specific patient satisfaction questionnaire. The results demonstrate high levels of patient satisfaction with private physiotherapy in Ireland, but raise some concerns regarding the cost of private physiotherapy treatment. Studies of patient satisfaction with similar processes of care internationally have previously reported high levels of satisfaction [7,30-33], but lack of consistent methodologies or outcome measures impedes direct comparison. The response rate of 55% (n = 131/240) and respondent characteristics were consistent with previous unsolicited postal surveys of patient satisfaction with healthcare who recorded an average response rate of 50% [20-22,37], and with studies used to develop 'patient satisfaction with physiotherapy' questionnaires [1,24-27,38,39].

Although the anonymous nature of the current study precluded the use of reminders, this should be employed in further studies to maximize response rates including the use of handwritten envelopes, the provision of free post address envelopes, postcard reminders, use of telephone reminders and provision of a questionnaire at final reminder stage [16,29,40-43]. However, results from the current study should be interpreted with caution notwithstanding the need for further evaluation of the data quality, internal consistency, reliability and validity of this questionnaire in an Irish population to assess cross cultural psychometric equivalence [49,50]. The authors also recommend that cross cultural equivalence (linguistic and psychometric) is assessed in other countries that intend to utilise this questionnaire [28,49-51], using sufficiently large sample sizes, where sample size of approximately five cases per item are advised to allow factor analysis to be valid, requiring up to 170 cases [48], which is consistent with the sample sizes used in the three phase psychometric evaluation during the development of the questionnaire [24]. Larger sample sizes of up to 10 cases per item may be required for valid logistic regression analysis, requiring up to 340 cases [48], and this should be considered in future studies.

Furthermore, the degree to which the results can be generalized to all patients receiving private physiotherapy in Ireland may be limited by the use of a sample of convenience in the selection of the physiotherapy practices, and by physiotherapist choice in patient selection, where despite provision of a study protocol for patient-selection, they may have selected patients whom appeared to be more satisfied with treatment [6,15,16,24]. This is acknowledged as limitation of the current study and the authors recommend that future studies should attempt to seek follow-up from a sample of non-respondents or those who fail to complete treatment as these patients may be more dissatisfied than respondents [6,15,24].

A systematic review of the methodologies of patient satisfaction measurement (interview or mail survey etc) [16] showed that although response rates for interviewer methods (telephone or face to face) are approximately 30% higher than mail surveys, the former tended to be more expensive and time consuming and that there was a tendency for patients to express gratitude and satisfaction with the service being provided (acquiescence bias). Although not as prevalent in mails surveys, this potential could have been minimised in this cross sectional survey if an independent researcher rather the treating physiotherapist had administered the questionnaire. Response bias may be an issue in questionnaires which offer Yes/No answers or fail to include negatively phrased questions, and can result in patients answering all questions similarly without attention to the question asked, giving rise to false positive results. [5,6,16]. This was minimised by using a questionnaire, which used a 5 point scale and included negatively phrased questions [6,16,24].

For the current study, interpretation was based on concordance with the optimum scores provided by Roush & Sonstroem [24] for each domain. The PTOPS questionnaire [24] establishes four domains with independent summary scores, and while the results for Enhancer and Location domains were comparable to the optimum scores, respondents were somewhat dissatisfied with Detractor issues and very dissatisfied with Cost domains, despite the fact that the average cost of €40 per treatment reflects current charges for private physiotherapy in Ireland, suggesting there are 'value for money' issues with private physiotherapy in Ireland. In fact, although not explored in this study, it is possible that the delayed access to public physiotherapy services in Ireland may have forced some respondents to unwillingly self fund their physiotherapy to ensure prompt access to care [12].

As this was the first time that this questionnaire had been used in a non-American population, supplementary socio-demographic and treatment data were gathered to examine their potential influence on satisfaction scores. Some studies have suggested that patient satisfaction may be associated with patient characteristics such as age, gender or educational status [16,34,35]. However, although multivariate analysis in the current study suggested that patient satisfaction was associated with older patients and those with spinal problems, this analysis predicted less than 10% of variance in the levels of satisfaction. Thus, it is possible that other patient characteristics such as mechanism of injury, chronicity, or clinical outcomes may influence satisfaction levels [16], and this is currently being investigated in a randomised controlled trial of private and public physiotherapy for patients with low back pain in Ireland [44]. Alongside this, the above recommendations regarding sample size and sample selection (including the follow up of patients who fail to complete treatment), questionnaire design and delivery methods, should be considered in future studies, to ensure that the widest possible range of patient satisfaction levels are recorded. This may minimise the lack of variability of patient satisfaction levels, which at present is typically high [7,30-33], thus improving the identification of definitive predictors in future regression or correlation analyses.

It has been reported that satisfied patients will return for treatment when the need arises, and will speak in favourable terms about the treatment and facility [38]. Thus, it is vital that private physiotherapists make efforts to ensure that their patients are satisfied. The treatment and patient details section of the survey instrument yielded information that may be useful to private physiotherapists from a business perspective. For example, the majority of patients learned about the physiotherapy practice through friends and former patients of the clinic emphasising word of mouth as a mean of attaining business, and only a fraction of patients learned about the clinic through the 'Golden pages business directory', indicating that the expensive cost of advertising may be unwarranted. Also the majority of patients attending physiotherapy presented with spinal complaints, and information of this nature may help to guide physiotherapists regarding advertising or continuing professional development (CPD) priorities.

Finally, the survey instrument also provided information regarding the number and cost of treatments, methods of payment and treatment approaches. The most common treatments received by patients were manual therapy, exercise therapy, advice and information and home exercises programmes (HEP's), which are supported in the current literature [45-47] where best practice supports a multimodal physiotherapy approach in addition to patient self-management through information, advice and HEP's for musculoskeletal conditions.

To summarise, this survey has measured for the first time the levels of patient satisfaction with private physiotherapy for patients with musculoskeletal pain in a non-American healthcare setting, finding high satisfaction levels in the Irish sample surveyed, and has confirmed a positive association between age and patient satisfaction with physiotherapy treatment. The study has provided a greater understanding and knowledge base for physiotherapy related satisfaction issues, which should encourage the routine measurement of patient satisfaction by practitioners and researchers in other European countries, and has the potential to assist physiotherapists in making choices regarding continuing professional development and marketing strategies, in a manner that incorporates patient feedback. However, prior to the use of this questionnaire in Ireland or indeed in Europe, further studies with sufficiently large sample sizes are necessary to evaluate the data quality, scale structure, reliability and validity of this survey instrument in different populations [28,48-51].

## Conclusion

### What is already known about this topic?

Patient satisfaction with physiotherapy treatment is an increasingly important patient-centred outcome, which is regularly overlooked or poorly measured in healthcare research using non validated measurement tools. The majority of published literature refers to American populations and no suitable validated measure of 'patient satisfaction with physiotherapy treatment' exists for European populations. Levels and determinants of patient satisfaction with physiotherapy treatment, such as patient characteristics, diagnosis or treatment outcome are not well understood.

### What does this article add?

This study reviewed the literature regarding patient satisfaction with physiotherapy treatment including existing 'patient satisfaction with physiotherapy treatment' questionnaires and described the selection of the "Physical Therapy Outpatient Satisfaction Survey" (PTOPS), it's translation from an American to a European version, and confirmed the feasibility of it's use with an Irish population. Results showed high levels of overall satisfaction with the service received, but some dissatisfaction regarding the cost of treatment.

## Competing interests

The author(s) declare that they have no competing interests.

## Authors' contributions

SC-F made substantial contributions to conception and design of the study, the acquisition, analysis and interpretation of the data, drafting of the manuscript and has given final approval of the version to be published. MP made substantial contributions to the acquisition, analysis and interpretation of data, was involved in the drafting the manuscript, and gave final approval of the version to be published. FD made substantial contributions to the acquisition, analysis and interpretation of data, was involved in the drafting the manuscript and gave final approval of the version to be published. SR made substantial contributions to conception and design of the study, ethical approval application preparation, revised it critically for important intellectual content and gave approval of the version to be published. MC made substantial contributions to conception and design of the study, ethical approval application preparation, revised it critically for important intellectual content and gave final approval of the version to be published. DH made substantial contributions to conception and design of the study, ethical approval application preparation, revised it critically for important intellectual content and gave final approval of the version to be published.

## Pre-publication history

The pre-publication history for this paper can be accessed here:


